# Physical activity-related injuries of college students in southern China: A 1-year prospective study

**DOI:** 10.1038/s41598-020-64317-5

**Published:** 2020-04-28

**Authors:** Dongchun Tang, Weicong Cai, Yang Gao, Shangmin Chen, Liping Li

**Affiliations:** 1 Injury Prevention Research Center, Shantou University Medical College, No.22 Xinling Road, Shantou, 515041 Guangdong, China; 2 Department of Non-Communicable Disease Control and Prevention, Shenzhen Center for Chronic Disease Control, Shenzhen, 518020 Guangdong, China; 3 Department of Sport and Physical Education, Hong Kong Baptist University, Kowloon Tong, Hong Kong China

**Keywords:** Epidemiology, Lifestyle modification

## Abstract

This prospective study was to describe the incidence and characteristics of physical activity-related injuries (PARI) among college students in southern China. Online questionnaires and telephone interviews were combined to investigate the physical activity (PA) participation and PARI occurrences every two weeks. Totally, 84 college students (men: 49; women: 35) with a mean age of 19.4 years completed the entire 1-year follow-up. Overall, 14250.3 PA exposure hours were collected and 36 PARI episodes were reported by 26 students, with an injury incidence density of 2.53 injuries per 1000 PA exposure hours and an injury risk of 0.43 injuries/student/year. July to September accounted for a highest proportion of injuries and half of the injuries happened in the evening. The majority of injuries occurred outdoors, happened in non-contact activities, were acute and involved the lower limbs, with sprains and strains being the primary injury types. Of all injuries, 80.6% resulted in PA withdrawal immediately and 58.3% led to absence from the next planned PA. No significant difference was found between men and women. This study reveals the problem of PARI among college students, which provides the direction for the prevention of PARI in this population.

## Introduction

The level of physical inactivity is rising in many countries, adversely affecting the health condition of the population and increasing the prevalence of non-communicable diseases^1^. Physical inactivity is now considered the fourth leading cause of death worldwide^1^. Conversely, participation in regular physical activity (PA) creates a range of benefits, including physical fitness, mental health, and the reduced risk of premature death and chronic diseases^2^. World Health Organization (WHO) established the importance of regular PA and published the Global Recommendations on Physical Activity for Health in 2010^1^. However, despite the benefits derived from regular PA, it is accompanied by the risk of adverse consequences—physical activity-related injuries (PARI)^3,4^. Negative consequences resulted from PARI were reported, such as financial burden and absence from PA participation and school lessons^5,6^. In addition, a history of injury was also identified as a significant risk factor for subsequent injuries^7^. Previous studies concerning the epidemiology of PARI among collegiate athletes showed that the injury incidence density varied from 1.91 to 11.7 per 1000 exposure hours^6,8,9^. This implies that the injury incidence density is relatively high and therefore, effective injury prevention programs are necessary to be developed with a first step to obtain epidemiological information about the epidemiology of PARI in targeted population^10^.

Previous researches on the epidemiology of PARI were conducted mainly through the cross-sectional study or injury surveillance program^4,6,9^. Moreover, most studies only reported the occurrences of injuries, while ignoring the calculation of injury incidence density (an important injury indicator)^11^. What was worse, the PA exposure time used to estimate injury incidence density in some reports was collected only once by asking the participants to recall their duration of PA engagement in a long period (e.g. 1 year), which would lead to the limitations of capturing its dynamics and ignoring the negative influence of PARI on PA participation during the study period^12^. Thus, a prospective study taking PA exposure time into account is urgent. Compared with primary and secondary school students, college students may have more chances to take part in more different kinds of PAs as they have less academic pressure after passing the college admission examination^13^. Additionally, school boarding frees college students from parental supervision and this allows them to participate in relatively risky PAs that are not allowed before^14^. Besides that, the continuous increase of physical education in universities and more sports facilities and venues provided for free on campus play a positive role as well. All these complies with the physically active lifestyle advocated by the Chinese government in the past decades. Previous study has revealed that the majority of college students in southern China were physically active according to the WHO’s recommended PA guidelines for adults^15^. However, there is a knowledge gap on the problem of PARI among this population. This is a barrier to develop effective injury prevention if we cannot have a good understanding of PARI for the targeted population^10^.

Collectively, we therefore carried out a 1-year prospective study among college students in southern China, a part of a series of studies funded by the National Natural Science Foundation of China, aiming to investigate the injury incidence density and risk, characteristics (time, circumstance, mechanism, body part, type, PA involved, consequence and severity) of PARI.

## Methods

### Study population

The study participants were recruited from the baseline survey (a two-stage study) conducted by our research team. The details of this two-stage study have been previously described^15^, and are summarized as follows: In the first stage, 2123 college students from two universities (one normal university and one comprehensive university) in Chaoshan district, southern China were recruited by the method of cluster random sampling in March and April 2017. In the second stage, 434 students who consented to be followed up were invited to complete the face-to-face interviews in April and May 2017. After the baseline survey, 101 participants from Shantou University (a comprehensive university) were asked if they were willing to participate in the follow-up study. A total of 88 students gave positive response and were initially recruited in September 2017. During follow-up period (September 2017 to September 2018), if no response to the follow-up questionnaires was received from the consenting participants, they would be excluded from this study. Finally, 84 participants completed the whole 1-year follow-up with a completion rate of 95.5%. The students met the following inclusion criteria were included: a) participation in the baseline study; b) 1st to 3rd grade students; c) ability to engage in PA; d) agree to sign informed consent for participation in the study.

### Data collection

The basic demographics were investigated in the baseline surveys during March to May 2017^15^, consisting of questions regarding age, sex, height, weight, study major, study year, living in a school dormitory (yes or no), suffering any diagnosed chronic disease/symptom (yes or no) and being a sports team member (yes or no). The information of screen time, PA participation and PARI occurrences were collected in the follow-up online questionnaire every two weeks from September 2017 to September 2018.

Screen time included the average daily duration of telephone and computer usage (hours/day) in the past two weeks, which was asked by “In the past two weeks, how long did you spend on computer or telephone per day?”

The International Physical Activity Questionnaire (IPAQ)^16^ and the Chinese version of the Children’s Leisure Activities Study Survey (CLASS-C)^17^ were combined and adapted to evaluate students’ PA participation per typical week in the past two weeks, which showed good reliability in our study (Cronbach’s alpha = 0.819). The participants were asked by “In the past two weeks, what kind of PA and its frequency and duration did you do that lasted at least 10 minutes each time on both a weekday and a weekend, respectively?” The weekly PA exposure time (cumulative hours per week) of each student was obtained by adding up the exposure time of each kind of PA (calculating by frequency multiplied by duration for each type of PA), and then the total PA exposure time in the 26 follow-up investigations was estimated accordingly.

PARI occurrences was ask by “In the past two weeks, did you suffer from injury that met at least one of the following criteria when undertaking PA^18^: you a) have to stop the current PA immediately and/or; b) cannot participate in the next planned PA and/or; c) cannot go to the class next day and/or; d) have to seek medical attention (including first-aid, seeing a doctor or receiving physical therapy, but excluding those using bandages only).”

Those students experienced PARI in the past two weeks were further interviewed by telephone to fill out the injury registration form, which was comprised of the detailed characteristics of PARI including time, circumstance, mechanism, type, body part, PA involved and consequence. The acute injury was defined as an injury that was cause by a specific, single event or had a sudden onset, while the overuse injury was defined as an injury that resulted from repeated microtrauma without an identifiable event related to the injury^19^.

### Procedures

At the beginning, explanatory statements of the study were distributed to 101 students who participated in our previous cross-sectional studies and informed consent forms were obtained from 88 participants. The hyperlink of follow-up questionnaire was subsequently sent to the consenting students by social networking or text message in an interval of two weeks from September 2017 to September 2018 (a total of 52 weeks). Each student would receive an automatically generated reminder message one day after the hyperlink was sent, reminding them to complete the online questionnaire. If the respondents did not fill out the questionnaires one day after receiving the reminder message, then they would be reminded again by telephone calls by our trained investigators. Those students who were still unwilling to complete the questionnaires after the telephone calls would be excluded. Finally, a total of 4 students dropped out due to their refusal to participate in the follow-ups.

Students who reported in the online questionnaire that they experienced PARI in the past two weeks were interviewed by telephone calls to verify if it met the PARI definition and criteria. If yes, those injured students were further interviewed to complete the injury registration forms. Considering that this study would last for one year, incentives were provided for the participants. After each follow-up survey, the phone charge of 2 yuan (equates to 0.285 USD) was given to each respondent who filled in the online questionnaires. Moreover, those injured respondents who completed the injury registration forms were get an extra phone charge of 3 yuan (equates to 0.428 USD).

The data were reliability-tested and validated by telephone interviews among all injured students one week after completing their follow-ups, and it turned out to be reliable (kappa coefficient = 0.735 ± 0.237).

### Ethics approval

This study was strictly carried out in accordance with the Declaration of Helsinki and the protocol was approved by Shantou University Medical College Ethics Committee (SUMC-2016-22).

### Statistical analysis

The power of the sample was conducted by G.Power software for power and sample size calculation. The constant proportion is the incidence of PARI in college students, which was obtained from our previous survey. The values of α, sample size and effect size were set as 0.05, 84, and 0.3, respectively for post-hoc analysis, and the corresponding power (1-β) of 0.9994598 was calculated. This indicates that the power (1-β) of this study is greater than 0.95. The values of α, power (1-β) and effect size were set as 0.05, 0.95, and 0.3, respectively for prior analysis, and the calculated sample size was 35. This indicates that the sample size of this study (n = 84) meets the minimum sample size requirement.

Continuous variables were described as mean and standard deviation (SD), while categorical variables were presented as number and percentage. Independent-sample *t* tests and Fisher’s exact tests were used accordingly to test the differences between men and women. The injury risks were calculated by dividing the total amount of injuries by the total number of students. The injury incidence densities were defined as the total amount of injuries divided by the total number of PA exposure hours during one-year period and determined per 1000 hours of exposure. The 95% confidence intervals (95% CIs) of the injury incidence density were calculated assuming a Poisson distribution. When the 95% CIs did not overlap, two injury incidence densities were considered significantly different^20^. The severity of injuries was subdivided according to the time loss of normal PA participation: mild (no time loss from PA), moderate (1–7 days of absence from PA) or sever (>7 days of absence from PA).

All statistical analyses were conducted using SPSS 23.0 (SPSS Inc. Chicago, IL, USA) and a two-tailed *P*-value less than 0.05 was considered statistically significant.

## Results

### Injury risk and injury incidence density

The mean age of 84 participants (men: 49; women: 35) was 19.4 years at entry (SD: 0.89; range: 18–21). A total of 36 PARI episodes were reported by 26 participants (31.0%, men: 16; women: 10), resulting in an overall injury risk of 0.43 injuries/student/year (men: 0.51; women: 0.31) (Table 1). The majority of the injured students suffered from one PARI (69.2%, 18/26), six sustained two PARI (23.1%, 6/26) and two experienced three PARI episodes (7.7%, 2/26) over the study period.

**Table 1:** Injury risk and injury incidence density of college students.

Variable	Total (n = 84)	Men (n = 49)	Women (n = 35)
Injured students	26	16	10
PARI episodes	36	25	11
PA exposure time (hours)	14250.3	8108.1	6142.2
Injury incidence	31.0%	32.7%	28.6%
Injury risk	0.43	0.51	0.31
Injury incidence density (95% CI)	2.53 (1.82–3.51)	3.08 (2.08–4.56)	1.79 (0.99–3.23)

The average PA exposure time was 3.26 hours/student/week (men: 3.18; women: 3.37), which amounted to a total of 14250.3 hours for the entire follow-up period (men: 8108.1; women: 6142.2). The overall injury incidence density was calculated as 2.53 injuries per 1000 PA exposure hours (95% CI, 1.82–3.51), with non-significant higher injury incidence density among men (3.08, 95% CI, 2.08–4.56) than women (1.79, 95% CI, 0.99–3.23) (*P* > 0.05) (Table 1).

### Time and circumstance of PARI

As presented in Table 2, July to September were the most vulnerable months for college students to sustain PARI (38.9%, 14/36). Half of the injuries occurred in the evening (50.0%, 18/36) and 25.0% (9/36) in the afternoon and morning, respectively. In addition, nearly seven out of ten (69.4%, 25/36) of injuries occurred outdoors. There was no significant difference between different sex in terms of time and circumstance of PARI (all *P* > 0.05).

**Table 2:** Time and circumstance of PARI among injured students.

Characteristics	Total n (%)	Men n (%)	Women n (%)	*Fisher’s Exact*	*P*-value
Month				4.043	0.225
January-March	3 (8.3)	1 (4.0)	2 (18.2)		
April-June	9 (25.0)	6 (24.0)	3 (27.3)		
July-September	14 (38.9)	9 (36.0)	5 (45.5)		
October-December	10 (27.8)	9 (36.0)	1 (9.0)		
Time				1.565	0.514
00:00–05:59	0 (0.0)	0 (0.0)	0 (0.0)		
06:00–11:59	9 (25.0)	5 (20.0)	4 (36.4)		
12:00–17:59	9 (25.0)	6 (24.0)	3 (27.2)		
18:00–23:59	18 (50.0)	14 (56.0)	4 (36.4)		
Circumstance				0.000	1.000
Indoors	11 (30.6)	8 (32.0)	3 (27.3)		
Outdoors	25 (69.4)	17 (68.0)	8 (72.7)		

### Mechanism and Type of PARI

Appropriately three-fifths of the injuries were acute (58.3%) and the rest were overuse (41.7%), and no significant difference was observed between men and women (*Fisher’s Exact* = 0.004, *P* = 0.951). The primary type of injury was joint/ligament sprains (33.3%), followed by muscle/tendon strains (27.8%) and abrasion (19.4%), and the similar distribution in different sex was observed (*Fisher’s Exact* = 5.112, *P* = 0.556) (Table 3).

**Table 3:** Mechanism and Type of PARI among injured students.

Characteristics	Total n (%)	Men n (%)	Women n (%)	*Fisher’s Exact*	*P*-value
Mechanism of PARI				0.004	0.951
Overuse	15 (41.7)	11 (44.0)	4 (36.4)		
Acute	21 (58.3)	14 (56.0)	7 (63.6)		
Type of PARI				5.112	0.556
Sprains	12 (33.3)	9 (36.0)	3 (27.3)		
Strains	10 (27.8)	7 (28.0)	3 (27.3)		
Abrasion	7 (19.4)	5 (20.0)	2 (18.2)		
Contusion	3 (8.3)	2 (8.0)	1 (9.1)		
Fracture	2 (5.6)	0 (0.0)	2 (18.2)		
Tendonitis	1 (2.8)	1 (4.0)	0 (0.0)		
Sunburn	1 (2.8)	1 (4.0)	0 (0.0)		

### Body parts of PARI

Amongst the 36 injury episodes, multiple locations of injury occurred in seven (four were sustained to two body parts, two were to three body parts and one was to four body parts), resulting in 47 injured body parts totally. Overall, 55.2% of all injuries occurred to lower limbs, 23.4% happened to upper limbs, 12.8% were located at head, neck and face, and the rest (8.6%) involved trunk. In terms of the specific injured body parts, ankle/foot was the most common sites (31.8%), followed by knee/calf (17.0%), shoulder/upper arm (8.5%) and wrist/hand (8.5%). The distribution of the injured body parts was comparable in men and women (*Fisher’s Exact* = 3.911, *P* = 0.238) (Table 4).

**Table 4:** Distribution of injured body parts among injured students.

Characteristics	Total n (%)	Men n (%)	Women n (%)
**Lower limbs**			
Ankle/Foot	15 (31.8)	9 (26.6)	6 (46.1)
Knee/Calf	8 (17.0)	5 (14.7)	3 (23.1)
Hip/Thigh	3 (6.4)	2 (5.9)	1 (7.7)
Sub-total	26 (55.2)	16 (47.2)	10 (76.9)
**Upper limbs**			
Shoulder/Upper arm	4 (8.5)	3 (8.8)	1 (7.7)
Elbow/Forearm	3 (6.4)	3 (8.8)	0 (0.0)
Wrist/Hand	4 (8.5)	3 (8.8)	1 (7.7)
Sub-total	11 (23.4)	9 (26.4)	2 (15.4)
**Trunk**			
Lower back	2 (4.3)	1 (2.9)	1 (7.7)
Chest	2 (4.3)	2 (5.9)	0 (0.0)
Sub-total	4 (8.6)	3 (8.8)	1 (7.7)
**Head/Neck/Face**	6 (12.8)	6 (17.6)	0 (0.0)
Total	47 (100.0)	34 (72.3)	13 (27.7)

### Physical activity involved

As presented in Table 5, more injuries occurred during playing basketball (25.0%), followed by gymnasium activities (16.6%) and walking (16.6%). No significant difference was found in PA items involved in PARI between different sex (*Fisher’s Exact* = 13.954, *P* = 0.079). Non-contact activities led to more than twice as much injuries as contact activities (69.4% vs. 30.6%), but no significant difference was found between different sex (*Fisher’s Exact* = 1.214, *P* = 0.439).

**Table 5:** Physical Activity involved in PARI among injured students.

PA involved	Total n (%)	Men n (%)	Women n (%)
**PA items**			
Basketball	9 (25.0)	7 (28.0)	2 (18.2)
Gymnasium activities	6 (16.6)	6 (24.0)	0 (0.0)
Walking	6 (16.6)	2 (4.0)	4 (36.3)
Running	5 (13.9)	4 (8.0)	1 (9.1)
Cycling	3 (8.3)	1 (16.0)	2 (18.2)
Jumping	2 (5.6)	2 (4.0)	0 (0.0)
Climbing	1 (2.8)	0 (8.0)	1 (9.1)
Rugby	1 (2.8)	1 (0.0)	0 (0.0)
Ping-pong	1 (2.8)	1 (4.0)	0 (0.0)
Yoga	1 (2.8)	0 (4.0)	1 (9.1)
Soccer	1 (2.8)	1 (4.0)	0 (0.0)
**Type of PA**			
Contact	11 (30.6)	9 (36.0)	2 (18.2)
Non-contact	25 (69.4)	16 (64.0)	9 (81.8)

### Consequences and severity of PARI

Of 36 injury episodes, 29 (80.6%) resulted in a withdrawal of PA participation immediately and more than half (58.3%) led to an absence from the next planned PA. A quarter (25.0%) required first aid and 30.5% needed to be treated in the accident and emergency department (A/E) or non-A/E. There was no significant difference between men and women (*Fisher’s Exact* = 4.732, *P* = 0.440) (Table 6). The average time loss of PA participation as a result of PARI was 1.58 days (range: 0 to 14 days). Overall, the majority of injuries was mild (61.1%, 22/36), 36.1% (13/36) was moderate and 2.8% (1/36) was severe. No significant difference was found between men and women regarding injury severity (*Fisher’s Exact* = 1.117, *P* = 0.351).

**Table 6:** Consequences and severity of PARI among injured students.

Characteristics	Total n (%)	Men n (%)	Women n (%)
**Consequences of PARI**			
PA withdrawal immediately	29 (80.6)	21 (84.0)	8 (72.7)
Absence from the next planned PA	21 (58.3)	15 (60.0)	6 (54.5)
Class absence next day	1 (2.8)	0 (0.0)	1 (9.1)
Require first aid	9 (25.0)	5 (20.0)	4 (36.4)
Treatment in A/E	3 (8.3)	1 (4.0)	2 (18.2)
Treatment in non-A/E	8 (22.2)	5 (20.0)	3 (27.3)
**Severity of PARI**			
Mild (0 days)	22 (61.1)	14 (56.0)	8 (72.7)
Moderate (1–7 days)	13 (36.1)	10 (40.0)	3 (27.3)
Severe (>7 days)	1 (2.8)	1 (4.0)	0 (0.0)

## Discussion

This prospective study revealed that nearly a third of college students (31.0%) sustained at least one PARI during the study year, which was significant higher than our earlier cross-sectional survey (22.7%)^4^. This might be due to the fact that cross-sectional study depends greatly on participants’ memories which would result in an underestimation of injury incidence. On the contrary, this 1-year follow-up study enabled us to avoid such a limitation and track injury occurrences more precisely over time. The injury incidence density of 2.53 injuries per 1000 PA exposure hours and the injury risk of 0.43 injuries/student/year in our subjects were both lower than those observed in physical education teacher education (PETE) students^8,21^. This discrepancy could be explained by the divergence of injury definitions, study designs and targeted populations^8,21^. Consistent with other research^6^, this study showed that the injury incidence densities and injury risks did not differ in men and women. Numerous studies found that men tended to be more active and had a greater possibility to participate in aggressive and competitive PAs and this would lead them to expose to more PARI^22^. However, other reports showed that the lower level of exercise performances and muscle endurance would place women in a higher risk of PARI^23^. These mixing effects might be the plausible explanations of why no sex-differences in injury incidence density and risk were observed in our study. Besides that, the small sample size of this study might also play a role.

Our results showed that July to September accounted for the highest injury incidence over the course of the months. The climate in this period is quite pleasant, with moderate temperature and little rainfall in southern China, making it the perfect time for students to participate in PA. In addition, this period includes summer vacation. College students do not need to take many school lessons and thus have more free time to take part in different kinds of PAs. Collectively, the higher level of PA participation contributes to the increased risk of injuries. Therefore, it is obvious that more attention should be paid to PARI prevention when promoting students to participate in PA actively, especially during the summer vacation. Additionally, more injuries occurred in the evening. This might be due to the fact that students need to attend school classes during the day and their participation of PA might be primarily in the evening. The dim light in the evening would be related to a greater likelihood of injury exposures. Previous research has recommended that outdoor activities promote physical well-being such as reducing the prevalence of myopia^24^. However, in line with other study^25^, we found that more injuries occurred outdoors. This might be that PA with higher risk usually occur outdoors as opposed to indoors^26^. Moreover, compared with indoor environments, more uncontrollable factors in the outdoor like weather might have a contribution to the higher possibility of PARI occurrences as well^27^. Consequently, we should put great emphasis on this problem and develop effective programs to decrease the risk of injuries.

Consistent with previous research^15^, more than half of PARI were acute in this study. Earlier reports have shown that contact team sports such as basketball and football might lead to more acute injuries^9,28^, while non-contact personal activities like running and climbing were mainly associated with overuse injuries^29,30^. The higher incidence of acute injuries was corresponding to the result in the present study that playing basketball involved in the majority of injuries. As one of the most popular high-contact team sports in China, basketball is extremely competitive and aggressive^31^. The players have to complete a variety of actions like rotating, jumping, landing, running and contact with other players^32^. All of these were related to the occurrences of injuries. Though the lower incidence, we need to take overuse injury into consideration because the injured students might not realize it if they suffered from minor injuries in the beginning and keep on doing exercise. This would gradually worsen their injuries. Therefore, special preventive measures are warranted to reduce the incidence of both acute and overuse injuries.

With regard to the type of injuries, sprains and strains accounted for the greatest proportion (61.1%). Similar findings were observed in other sport-active populations^9,33^. Players who jump, leap, land and sprint are more susceptible to sustain sprains and strains and the injured might feel a sudden pain at the beginning and unable to stand or walk subsequently^34^. Additionally, the high likelihood of recurrence of sprains and strains could adversely affect the normal PA participation and even hinder students from PA involvement^21^. Several preventive measures such as improving the balance ability by special training programs, increasing the utilization of protective equipment or emphasizing the importance of warming-up exercises have been introduced to reduce these types of PARI^35,36^. Another distinctive characteristic is that more than half of the injuries occurred in the lower limbs, which was aligned with the reported studies^37^. More specially, ankle and foot were the most predominant injured body parts and accounted for nearly one-third of all injuries. This could be found in other reports focused on various sport-active populations^9,33^. These consistent findings might due to the dominant usage of lower limbs when undertaking PAs because actions like pushing, jumping and running preferentially involved in lower limbs^32^. Additionally, the anatomical characteristics and neuromuscular controls in lower limbs may also play a role^38^. For instance, the ankle and knee are free to bend or stretch and this might lead to the problem of excessive off-axis loadings and result in injuries^38^.

In the present study, more than four-fifth of injuries (80.6%) led to an absence from PA participation immediately and 58.3% of them resulted in a withdrawal time of the next planned PA. Furthermore, the majority of injuries were mild and moderate and the average time loss of PA participation due to injuries was 1.58 days. Though PARI episodes are primarily minor, we still need to fully considerate both short-term and long-term negative consequences because they would prevent students from physically active and achieving the purpose of maintaining optimal individually physical and mental health^21^. Except for the adverse effect on PA participation, PARI could cause other direct and indirect costs^5,6^. Nevertheless, researchers believe that most PARI could be preventable, and costs due to injuries would be reduced accordingly if effective injury-prevention programs could be developed and implemented^39^. Some limitations should be noted. Firstly, the participants who involved in this study were not selected completely randomly from the baseline survey and the sample size was small because not all students were willing to cooperate with such a long-time investigation. This may limit the representativeness and generalizability of our study somewhat. Moreover, we collected the PA exposure time in a limited period to measure accurately, but it was still likely to be overestimated since individuals were more likely to over-report their PA exposures out of social desires^40^. The overestimation of PA may result in the underestimation of injury incidence densities in this study. Therefore, future prospective study in larger sample size obtained from random sampling and PA measured objectively and subjectively are warranted. In addition, other relevant factors such as diet, psychological stress and socio-culture should be assessed due to their potential influence on risk of injury.

In conclusion, we observed that 31.0% of college students sustained at least one PARI during 1-year follow up period, with an overall injury incidence density of 2.53 per 1000 PA exposure hours and an injury risk of 0.43 injuries/student/year. July to September accounted for the highest injury incidence density and half of the injuries occurred in the evening. A great portion of injuries occurred outdoors, happened in non-contact activities, were acute and involved the lower limbs, with sprains and strains being the major types of injuries. Of all injuries, 80.6% resulted in PA withdrawal immediately and 58.3% led to the absence from the next PA. These above data reveal the problem of PARI and inform that injury-prevention programs are needed to reduce the occurrences of PARI among college students especially when stimulating them to be physically active.
